# 

**Published:** 1877-03

**Authors:** 


### Table of Contents.

### ORIGINAL COMMUNICATIONS.

On the Post-Partem Use of Ergot in Obstetrical Practice. *By Wm. Abram Love, M.D:, Atlanta* ........ 705

Cerebro-Spinal Meningitis. *By J. G. Westmoreland, M.D., Atlanta* 712

Traumatic Septicæmia. *By W D. Boozer, M.D., Hogansville, Ga* .. 715

Epilepsy from Disease of Internal Ear. *By R. W. Westmoreland, M.D., Atlanta* ........ 717

### REPORTS OF SOCIETIES.

Atlanta Academy of Medicine........ 719

### SELECTIONS.

Secondary Hemorrhages........ 729
So-called Ulceration of the Womb ........ 734
Treatment of Tracheal and Laryngeal Stenosis ........ 737

### EDITORIAL.

Advances in medicine........ 740

### BIBLIOGRAPHICAL.

A Treatise on the Science and Practice of Midwifery........ 743
The Tonic Treatment of Syphilis........ 744
Transactions of the American Medical Association ........ 746
A Report on the Influence of Climate on Pulmonary Diseases in Minnesota ........ 746
The Practitioner's Hand Book of Treatment........ 747
A Course of Practical Histology........ 747

### EXCERPTA.

A Marvelous Story of Inoculated Croup........ 747
On Eruptions (not Corymbiform) Produced by Nerve Influence........ 748
Treatment of Psoriasis by Rubber Sheeting........ 750
A New Remedy, Called Digestine........
New Method of Treating Urethral Strictures........ 752
Remarkable Case in Obstetrics ........ 753
Sand-Frictions in Cutaneous Diseases........ 754
Forcible Extension of Nerves in Traumatic Tetanus ........ 755
Osteotomy in "Genu Valgum"........ 756
"Opium Antidote" ........ 757
European Correspondence ........ 758
Index........ 765

